# Polygenic risk score for tumor aggressiveness and early-onset prostate cancer in Asians

**DOI:** 10.1038/s41598-022-17515-2

**Published:** 2023-01-16

**Authors:** Sang Hun Song, Eunae Kim, Yu Jin Jung, Hak-Min Kim, Moon Soo Park, Jung Kwon Kim, Hakmin Lee, Jong Jin Oh, Sangchul Lee, Sung Kyu Hong, Seok-Soo Byun

**Affiliations:** 1 Department of Urology, Seoul National University Bundang Hospital, 300, Gumi-dong, Bundang-gu, Seongnam-si, Kyunggi-do 463-707 South Korea; 2 Department of Urology, Seoul National University College of Medicine, Seoul, South Korea; 3 Procagen, Seongnam, South Korea; 4 Department of Medical Device Development, Seoul National University College of Medicine, Seoul, South Korea; 5 Seolleung Top Clinic, Seoul, South Korea

**Keywords:** Genetics, Biomarkers, Oncology

## Abstract

We attempted to assess the performance of an ethnic-specific polygenic risk score (PRS) designed from a Korean population to predict aggressive prostate cancer (PCa) and early-onset (age < 60). A PRS score comprised of 22 SNPs was computed in 3695 patients gathered from one of 4 tertiary centers in Korea. Males with biopsy or radical prostatectomy-proven PCa were included for analysis, collecting additional clinical parameters such as age, BMI, PSA, Gleason Group (GG), and staging. Patients were divided into 4 groups of PRS quartiles. Intergroup differences were assessed, as well as risk ratio and predictive performance based on GG using logistic regression analysis and AUC. No significant intergroup differences were observed for BMI, PSA, and rate of ≥ T3a tumors on pathology. Rate of GG ≥ 2, GG ≥ 3, and GG ≥ 4 showed a significant pattern of increase by PRS quartile (p < 0.001, < 0.001, and 0.039, respectively). With the lowest PRS quartile as reference, higher PRS groups showed sequentially escalating risk for GG ≥ 2 and GG ≥ 3 pathology, with a 4.6-fold rise in GG ≥ 2 (p < 0.001) and 2.0-fold rise in GG ≥ 3 (p < 0.001) for the highest PRS quartiles. Combining PRS with PSA improved prediction of early onset csPCa (AUC 0.759) compared to PRS (AUC 0.627) and PSA alone (AUC 0.736). To conclude, an ethnic-specific PRS was found to predict susceptibility of aggressive PCa in addition to improving detection of csPCa when combined with PSA in early onset populations. PRS may have a role as a risk-stratification model in actual practice. Large scale, multi-ethnic trials are required to validate our results.

## Introduction

Incidence of prostate cancer (PCa) is constantly on the rise, especially with introduction of nationwide early PSA screening, westernized diets with high dietary fat, and increase in average life expectancy^1^. Such increase is more marked in East Asian countries, where PCa is known to take on a more aggressive form^2^, which can be explained by both genetic predisposition and environmental effects^3^. However, conventional screening with PSA alone is vastly limited, due to ambiguities affecting PSA levels such as prostatitis or benign hyperplasia. This has fueled the need for additional biomarkers more specific to PCa, in order to tailor screening and intervention methods and aid clinical decision making process.

Interest in genetic association of PCa has rapidly grown with the finding of nominal mutations eligible for therapy, such as BRCA 1/2 that have been recently indicated for treatment with PARP (Poly-ADP ribose polymerase) inhibitors through major clinical trials^4^. However, as not all genetic background of PCa can be explained with the inheritance of high penetrance genes alone, research on the combinatory effect of small genetic variants or SNPs (single nucleotide polymorphisms) has gained popularity, with numerous positive results on different models utilizing the technique^5–7^. A recent multi-ethnic study found more than a fivefold increase in risk for any PCa and PCa-related deaths^8^.

While a handful of models with moderate predictive performance have been introduced using polygenetic risk in PCa, there is a disproportion in the level of evidence available for models to be used in Asian populations. Most models use datasets that consists of largely European heritage, leading to an overwhelming under-representation of Asian genetics, while many studies have underlined the difference in variants by ethnicity^9,10^. Also, while previous results have found significant findings for SNP-based prediction of any PCa^5^, whether such models can predict aggressiveness or early onset still requires further research. In this study, we aimed to test a polygenic risk score (PRS) model for prediction of high-risk, aggressive PCa and examine performance in patients with early age at diagnosis.

## Materials and methods

### Study population and genotyping

Data for this multicenter study was gathered from 4 tertiary medical centers (Seoul National University of Bundang Hospital, Seoul National University of Hospital, Chungbuk National University Hospital and the Catholic University St. Vincent hospital Seoul) and was Institutional Review Board approved at SNUBH (B-1607/355-302). Informed consent for study and genotyping was obtained for all subjects. Patients who had biopsy or pathology proven PCa through transrectal ultrasound prostate biopsy and/or radical prostatectomy (RP) were included for analysis. Healthy controls with genotyped data were also included. All patients were of Asian (Korean) ethnicity. Routine clinical parameters including age, BMI, PSA, Gleason Scores (grade groups), and clinicopathological staging were obtained. Genotyping was performed using the Korea Biobank array (K-CHIP) per manufacturer instructions with 200 ng of DNA from blood or saliva^11^. Clinically significant PCa (csPCa) was defined as Grade group (GG) ≥ 2. All methods included in this study was carried out in accordance with relevant guidelines and regulations.

### PRS model development

The PRS model described in a previous study^12^ were implemented for calculating genetic risk in the study participants. PRS is an aggregate sum of the effect size estimate or Cox proportional hazard ratio multiplied by the number of risk alleles found in the patient’s genotype^13^. Our PRS was developed using 10,187 genetic samples with 2702 PCa cases and 7485 controls, later validated with in an independent cohort with 311 cases and 822 controls, achieving a performance of AUC 0.700 (95% CI 0.667–0.734) with 29 top SNPs included. In this study, a 22 SNP model with reported AUC of 0.689 (95% CI 0.655–0.723) in csPCa were included after filtering excess SNPs with inadequately low allele frequencies or call rates (Supplementary Table 1). A single SNP (rs72725879) was replaced with the next highest performing variant (rs1456315) in the same LD block (Locus *PRNCR1*) due to replication inconsistencies when constructed as a custom array. All candidate SNPs during initial development were chosen only if proven on association studies in previous literature. Details on model development can be found in a previous article^12^.

### Statistical analysis

Patients were divided into 4 groups based on calculated PRS by quartile (0–24.9, 25–49.9, 50–74.9, 75–100th percentile). Subsets were further assessed for intergroup differences in GG distribution as well as pathologic grade, using student t-tests, Pearson Chi, and ANOVA statistics for continuous, categorical, and multi-categorical variables. Overall model predictive performance was evaluated using Area Under Receiver Operating Characteristic (AUROC, AUC). Logistic regression analysis was performed for PCa risk. All statistical analyses were performed using IBM SPSS software package version 25.0 (Statistical Package for Social Sciences™, Chicago, IL, USA).

## Results

Total 3695 patients were included in final analysis, of whom 3203 were PCa patients and 483 controls. Mean age for the overall sample population was 67.3, with mean PSA of 29.8 ± 192.2 ng/ml (Table 1). 132 patients (4.1%) harbored GG1 tumors overall, and others comprised of 26.8% GG2, 41.1% GG3, and 28.0% > GG4. 2170 patients (58.7%) underwent RP for csPCa, with 823 patients harboring high stage tumors (≥ pT3a) at final pathology. When divided by PRS quartiles, no significant intergroup differences were observed for BMI, PSA, and rate of high stage tumors at RP pathology. Only age between the 25–49.9th percentile and 75–100th percentile was found to be significant (p = 0.036), with no other notable intergroup outcome distinctions.

**Table 1:** Baseline clinical and pathologic characteristics.

Groups by percentile	All	< 25	25–50	50–75	≥ 75	p-value
Age (years), mean ± SD	67.3 ± 7.6	67.1 ± 7.5	67.8 ± 7.7	67.5 ± 7.6	66.8 ± 7.6	0.036
BMI (kg/m^2^), mean ± SD	24.5 ± 2.7	24.6 ± 2.7	24.4 ± 2.8	24.4 ± 2.5	24.7 ± 2.9	0.435
PSA (ng/ml), mean ± SD	29.8 ± 192.2	19.0 ± 63.6	25.1 ± 109.2	37.7 ± 206.9	37.0 ± 294.6	0.116
**Grade group (%)**						0.052
1	132 (4.1)	44 (4.8)	36 (3.9)	31 (3.4)	21 (2.3)	
2	857 (26.8)	182 (19.7)	227 (24.6)	223 (24.1)	225 (24.4)	
3	1316 (41.1)	270 (29.2)	319 (34.5)	340 (36.8)	387 (41.9)	
4	409 (12.8)	87 (9.4)	101 (11.0)	115 (12.4)	106 (11.4)	
5	487 (15.2)	108 (11.7)	120 (13.0)	130 (14.1)	129 (14.0)	
Pathologic ≥ T3a (%)	823 (22.5)	176 (19.3)	223 (24.4)	210 (22.9)	214 (23.3)	0.080

*SD* standard deviation, *BMI* body mass index, *PSA* prostate specific antigen.

**Figure 1:**
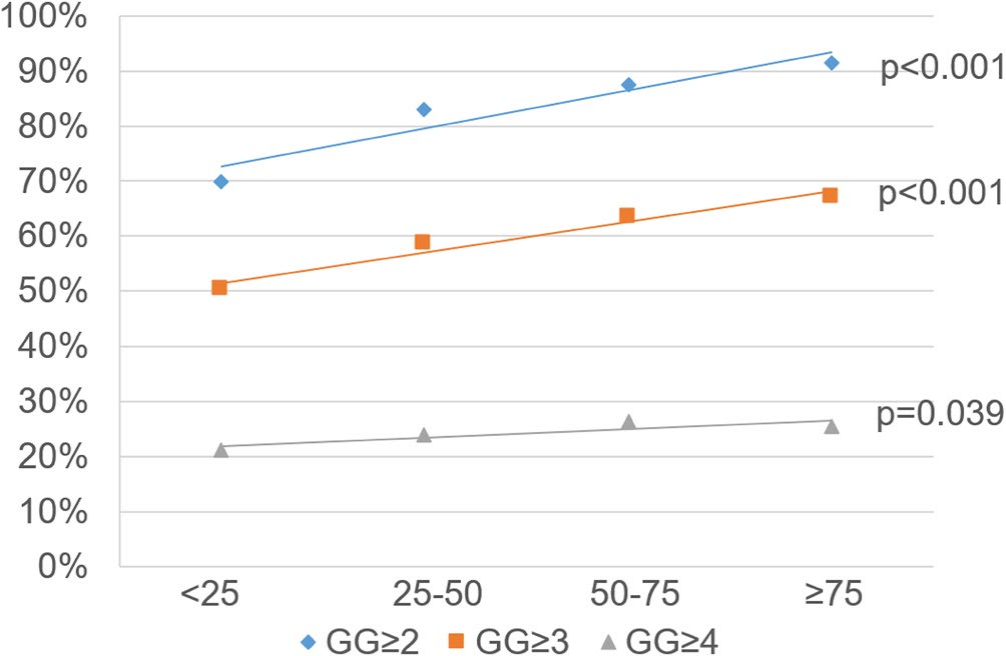
Percentage of GG ≥ 2, GG ≥ 3, and GG ≥ 4 histopathology by PRS percentile.

However, rate of csPCa showed a sequential increase, from 70.0% patients harboring GG ≥ 2 in the first quartile, to 83.1%, 87.5%, and 91.6% in the 2nd, 3rd, and 4th quartiles, respectively (p < 0.001) (Fig. 1). Rate of intermediate to high-risk pathology showed a similar pattern, with GG ≥ 3 increasing from 50.3% in the bottom quartile to 58.5%, 63.4%, and 67.2% in the 2nd, 3rd, and 4th quartiles, respectively (< 0.001). Rate of GG ≥ 4 resembled a comparable increase in the first 3 quartiles from 21.1%, 23.9%, to 26.5%, but was slightly decreased in the highest quartile with 25.4% (p = 0.039).

When using the lowest PRS quartile as reference (< 25 percentile), all sequential higher PRS risk groups showed an increase in risk for GG ≥ 2 and ≥ 3 tumor pathology (Table 2). Patients with the highest genetic risk based on PRS (≥ 75 centile) had a 4.6-fold increased risk for clinically significant disease (95% CI 3.544–6.098, p < 0.001), as well as a 2.0-fold increased risk for more aggressive GG ≥ 3 tumors.

**Table 2:** Risk of GG ≥ 2 and GG ≥ 3 relative to the low-risk group (PRS < 25th percentile).

Groups by percentile	Risk of GG ≥ 2			Risk of GG ≥ 3		
	HR	95% CI	p-value	HR	95% CI	p-value
≥ 75	4.649	3.544–6.098	< 0.001	2.026	1.678–2.446	< 0.001
50–75	3.008	2.364–3.827	< 0.001	1.708	1.419–2.057	0.001
25–50	2.105	1.685–2.629	< 0.001	1.392	1.158–1.673	< 0.001
< 25	Reference	–	–	Reference	–	–

*GG* grade group, *HR* hazard ratio, *CI* confidence interval.

**Figure 2:**
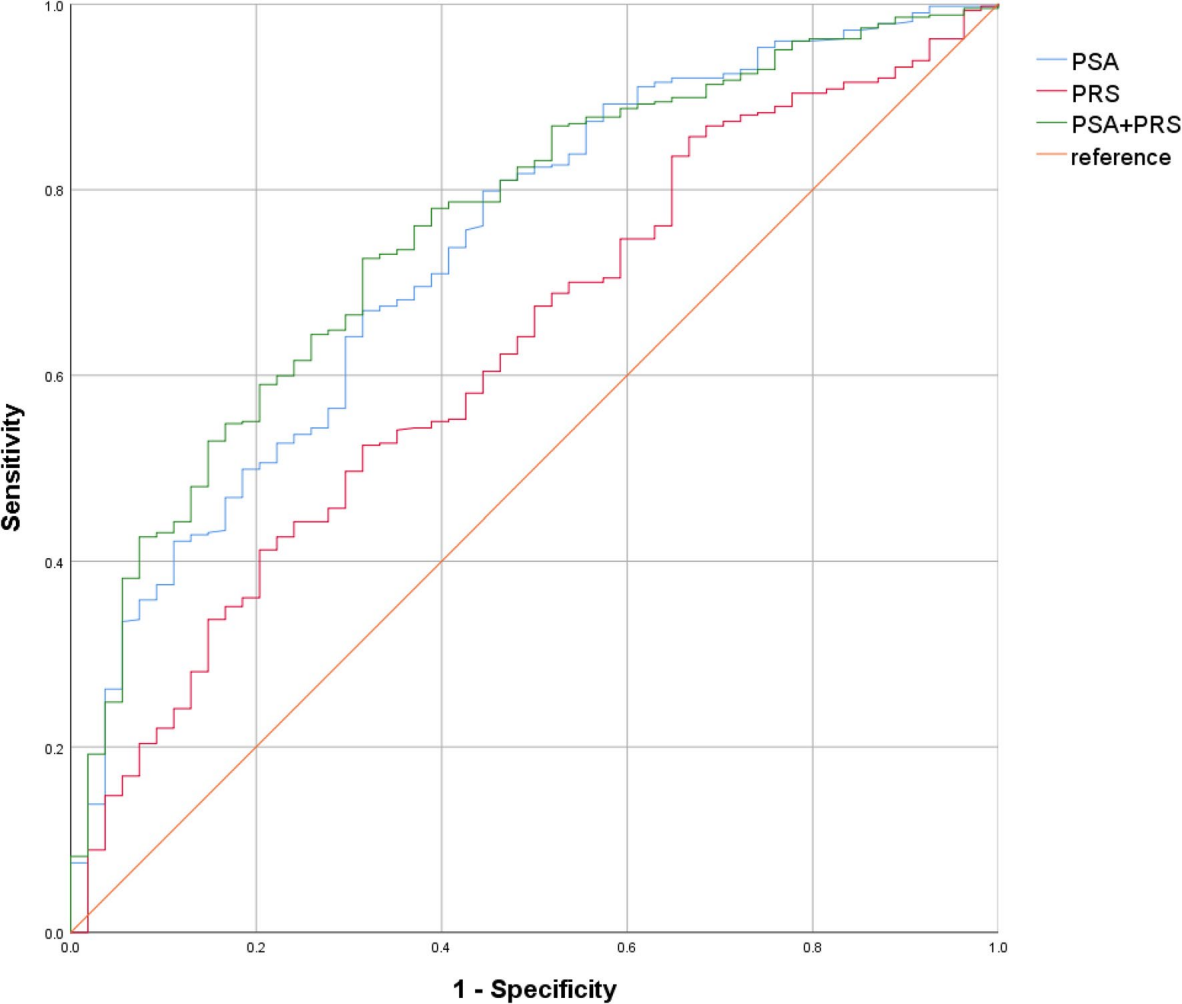
AUROC of PSA, PRS, and combined PSA + PRS model in early onset csPCa (≥ GG2 in age < 60).

PRS alone showed a moderate predictive performance of AUC 0.627 (95% CI 0.550–0.703, p = 0.002) for men with early-onset csPCa before the age of 60 (Fig. 2), with PSA outperforming PRS alone with AUC 0.736 (95% CI 0.666–0.805, p < 0.001). However, when combined with PSA, the clinicogenetic model of PRS + PSA outperformed either model with a AUC of 0.759 (95% CI 0.694–0.823, p < 0.001), suggesting the clinical utility of PRS for detection of early-onset disease requiring active intervention.

## Discussion

In this multicenter analysis of polygenetic risk for PCa, a PRS model constructed in a predominantly Asian population resulted in an increasing rate of high grade tumors detected at RP or biopsy in males with higher PRS. Patients with the highest genetic risk in the top quartile showed a 4.6-fold and 2.0-fold increase in GG ≥ 2 and GG ≥ 3 pathology, respectively. For men with early-onset disease (GG ≥ 2 prior to age 60), combination of PRS to the conventional PSA model improved predictive performance of AUC 0.759, from AUC 0.736 and 0.627 in PRS and PSA alone, respectively. These findings further support the feasibility and usefulness of genetic models for prediction and stratification of high risk patients based on germline mutation, especially via ethnic-specific models.

Previous studies on genomic risk from GWAS summary statistics laid strong foundations for any PCa risk but show mixed results on the ability to predict aggressive, intermediate-high risk disease^14^. A meta-analysis comprised of over 100,000 cases and 120,000 controls showed that a genetic score aggregate of total 269 risk variants was significantly associated with three to fivefold increase in absolute risk for PCa across ancestry, but was not able to differentiate aggressive disease defined as ≥ T3, regional N1/M1+, GG ≥ 4, or PSA ≥ 20 ng/ml^9^. The large model developed and validated in a Japanese-specific cohort found no difference in aggressive PC despite moderate performance of AUC 0.679 for any PCa^15^. A multiethnic dataset of approximately 50,000 cases and 30,000 controls^8^ described positive predictive performance of PRS to detect aggressive PCa, where HR for ≥ T3, regional N1/M1+, GG ≥ 2, or PSA ≥ 10 ng/ml was significantly higher in the top 20th percentile, with near 4.5-fold increase in the Asian subset. A more recent study in European men found a strong association for overall-risk increase for PCa based on PRS stratification, but not for high-grade tumors^16^. Other studies have produced similar results, with no strong association with tumor grade and aggressiveness^5,17^. As such, the results from our finding establishes potential for possible stratification for high-grade disease based on PRS, with sequential increase in GG ≥ 4 tumors being identified even within a case–control cohort largely composed of PCa patients. However, limitations of relatively few controls and overall small sample size, as well as lack of family history information must be addressed to fully explain the differences in tumor grade distribution in our study.

While patients diagnosed with PCa at a young age are evidently at risk for high penetrance pathogenic germline mutations such as BRCA1/2^18^ or HOXB13^19^, predicting earlier age of onset based on multiple variants with small effect size potential have produced ambiguous results, with a previous study^20^ finding a PRS of 24 SNPs to be discriminatory for any and high grade GG ≥ 2 PCa only between age 60–70 years, losing predictive value in younger patients, most likely due to low prevalence and high likelihood of low-grade disease in this age group. Recent results from a Japanese cohort of 4893 PCa cases^6^ found patients with high PRS to have a younger mean age at diagnosis by 2.7 years (68.7 vs. 71.4 years in the top and bottom 5%, respectively), but weakened statistical association between PRS and early onset PCa when discarding variants without significant association with Japanese PCa. On the other hand, Seibert et al. designed a PRS utilizing data from the PRACTICAL consortium and UK ProtecT study, and found that a score of 54 SNPs were significantly predictive of early age at diagnosis of aggressive PCa^5^. A meta-analysis in males of European ancestry identified a novel locus rs138004030 for age of onset ≤ 55^21^, adding to the list of risk alleles significant for diagnosis ≤ 50 years in predominantly Caucasian dataset^22^.

Discrepancies in PRS performance by ethnic groups is not new, due to obvious variations in genetics as well as sociocultural environments. As such, accurate estimation of genetic contribution to PCa development inevitably requires development of ethnic-specific optimization^9^. However, Asian populations are vastly under-represented in most GWAS summary statistics, limiting direct implementation and generalizability in different patient populations and few PRS currently developed^23^. In this study, a KoreanChip customized for optimal evaluation including rare and novel variants found in Korean patients were utilized^11^, improving coverage and better representing genomic variations in East Asians. In order avoid overfitting by inclusion of non-PCa associated SNPs with high statistical significance, only variants with literature evidence for PCa risk in East Asians were included for PRS development. Also, as sample size is critical to accurate estimation of variant risk in a population, this study has strengths by using one of the largest patient cohorts in an East Asian population to our knowledge.

To note, the mean age of patients included this study were not vastly different by PRS quartiles, with the only significant difference found between the top quartile and patients in the 25–50th percentile (Table 1). PSA was also not significant for intergroup difference when stratified by PRS, suggesting the potential clinical utility of PRS when used as a reflex test when PSA alone is limiting. In fact, the clinicogenetic model of PSA augmented with PRS showed best performance, suggesting the additive value in real-life scenarios when other PCa parameters such as DRE positivity, MRI findings, or other biomarkers (e.g. 4 K Score, prostate health index) are used in clinical decision making.

Our study is not without limitations. First, family history of PCa was not including for analysis. Familial PCa is undeniably important in PCa risk analysis, with a person with 2 first-degree relatives inflicted with PCa to be 5 times more likely to develop the disease during his lifetime^24^. However, in previous literature^5^, the predictive performance of a PRS did not improve nor was undermined by the inclusion and exclusion of family history, and may not have had a detrimental effect on our model. Second, only Korean populations ethnicities were included during analysis, and while this does reinforce tailoring to East Asian populations, further assessment for generalizability of the model is essential. In addition, while the effect size estimates for variants were calculated from a previous cohort including over 7000 healthy controls, fewer healthy controls were included in this study, as the intent was to evaluate its ability to discriminate high-risk subgroups within PCa populations. While enrolled patients have considerable overlap to our previous study and may have caused an overfitting bias, these results indicate that PRS has the potential to discriminate and stratify high-grade, aggressive tumors among csPCa. Also, routine clinical parameters such as prostate volume or MRI findings were not available for analysis, the inclusion of which could have enhanced model performance. Inquiry for role in treatment selection in specific subgroups such as patients eligible for active surveillance requires further research, especially in Asian populations where the criteria for enrollment is still unclear^25^ due to different tumor characteristics.

Despite these limitations, this study shows that a PRS can be readily exploited in practice for predicting aggressive tumors that may occur early on in males with high genetic risk based on SNP mutations. While PRS may be inadequate as a diagnostic tool, it holds strong potential for diversifying screening and intervention strategies depending on germline risk in the era of precision medicine.

## Conclusion

A PRS specific to Asian males was found to increase detection of aggressive tumors, with men in the top 25 percentile harboring more than fourfold risk for GG ≥ 2 and twofold risk for GG ≥ 3 pathology. PRS combined with PSA was able to better predict early onset, outperforming PSA alone. These results bolster the role of genetic testing in risk stratification in PCa patients and provide evidence for active implementation in early stages of patient screening and diagnosis. Future validation in multi-ethnic cohorts are required to broaden potential applications.

## Supplementary Information

Supplementary Table 1.
