# Supplementary material for: Polygenic risk score for tumor aggressiveness and early-onset prostate cancer in Asians
Source: Sci Rep. 2023 Jan 16;13:798. doi: 10.1038/s41598-022-17515-2 (PMC9842611; doi:10.1038/s41598-022-17515-2)
Supplement: Supplementary file 1 — Supplementary Table 1. [file 41598_2022_17515_MOESM1_ESM.docx]

**Supplementary Table 1. SNPs for PRS construction and respective ORs.**

| **SNP** | **Risk allele** | **Weighted OR** |
| --- | --- | --- |
| rs7591218 | A | 0.16299 |
| rs11125927 | G | 0.220741 |
| rs77167534 | C | 0.183202 |
| rs2242652 | G | 0.180204 |
| rs1983891 | T | 0.1441 |
| rs4711748 | T | 0.117783 |
| rs339331 | T | 0.25515 |
| rs6955627 | C | 0.128857 |
| rs1512268 | T | 0.24373 |
| rs1456315 | T | 0.538026 |
| rs10505477 | A | 0.130151 |
| rs4242384 | C | 0.565882 |
| rs10993994 | T | 0.149282 |
| rs11817544 | C | 0.177453 |
| rs140783917 | C | 0.290887 |
| rs10896449 | G | 0.339325 |
| rs56159348 | T | 0.178887 |
| rs7489409 | C | 0.193097 |
| rs8023793 | A | 0.177692 |
| rs11263763 | A | 0.357389 |
| rs2659124 | T | 0.168064 |
| rs2238776 | G | 0.117883 |
